# Outcomes of Bronchial Artery Embolization in Patients With Moderate to Severe Hemoptysis: A Longitudinal Study From a Tertiary Care Center

**DOI:** 10.7759/cureus.88273

**Published:** 2025-07-18

**Authors:** Prashant Sarda, Abhay Pratap Singh, Chaitanya Saradhna, Tejas Sood, Ritisha Bhatt, Kiran Kumar Shetty

**Affiliations:** 1 Radiodiagnosis, Shri Guru Ram Rai Institute of Medical and Health Sciences, Dehradun, IND; 2 Pulmonary and Critical Care Medicine, Shri Guru Ram Rai Institute of Medical and Health Sciences, Dehradun, IND; 3 Respiratory Medicine, Shri Guru Ram Rai Institute of Medical and Health Sciences, Dehradun, IND; 4 Product Performance and Engineering, Meril Life Sciences, Vapi, IND

**Keywords:** bronchial artery embolization, bronchiectasis, embox, polyvinyl alcohol, pulmonary hemorrhage

## Abstract

Background: Hemoptysis, particularly in its moderate to severe forms, is a life-threatening respiratory emergency with multiple underlying etiologies, including post-tuberculosis sequelae, bronchiectasis, and aspergilloma. Bronchial artery embolization (BAE) has emerged as a first-line, minimally invasive intervention for controlling bleeding, but long-term outcomes and recurrence rates vary across populations, particularly in tuberculosis-endemic regions.

Methods: This longitudinal observational study was conducted over 18 months at a tertiary care center, enrolling 203 patients aged 18 years or older presenting with moderate to massive hemoptysis. All patients underwent clinical evaluation, radiological imaging, and BAE using Embox (Meril Life Sciences Pvt. Ltd., Vapi, India). Demographics, etiology, vessel involvement, immediate and follow-up outcomes at one and three months, and complications were recorded. The primary endpoint was immediate clinical success (bleeding cessation within 24 hours), while secondary endpoints included recurrence, treatment failure, and complication rates.

Results: Moderate hemoptysis was observed in 119 (58.6%) patients, while massive hemoptysis occurred in 84 (41.4%). Aspergilloma was the most common underlying etiology, identified in 89 (43.8%) cases, followed by bronchiectasis in 39 (19.2%) and fibrocavitary lesions in 24 (11.8%). Bilateral bronchial artery involvement was seen in 64 (27.8%) patients. The overall immediate clinical success rate was 151 (74.3%), whereas recurrence or treatment failure occurred in 22 (10.8%) patients, and mortality was reported in seven (3.4%) cases. Postprocedural complications were common, with chest pain in 119 (58.6%) and fever in 30 (14.8%) patients. Severe neurological events were rare, occurring in only one patient (0.5%). Most complications were self-limiting and showed a declining trend over time.

Conclusion: BAE is an effective and relatively safe intervention for controlling moderate to massive hemoptysis, particularly in patients with post-tuberculosis sequelae. Although recurrence and minor complications are common, BAE offers a valuable treatment modality in both emergency and elective settings, especially for patients unfit for surgery.

## Introduction

Hemoptysis, the expectoration of blood originating from the inferior respiratory tract, is a potentially life-threatening ailment that requires prompt assessment and management. While mild episodes may be self-limiting, moderate to severe hemoptysis, commonly defined as expectoration of over 100 mL of blood in 24 hours, can lead to significant morbidity and mortality if not rapidly controlled [1]. The etiologies of hemoptysis are diverse and include infectious, inflammatory, vascular, and neoplastic causes. Among these, post-tuberculosis sequelae, bronchiectasis, and malignancy are often implicated, mainly in regions with high tuberculosis prevalence [1].

Bronchial artery embolization (BAE) has arisen over the past two decades as the preferred interventional radiological treatment for moderate to massive hemoptysis. It is advantageous for patients who are poor surgical candidates or those in whom immediate surgical control is not feasible. The procedure involves the selective catheterization of bronchial and sometimes nonbronchial systemic arteries, followed by embolization using various agents, such as polyvinyl alcohol (PVA) particles, coils, or gelatin sponges. The goal is to achieve hemostasis while preserving surrounding healthy tissue and minimizing complications [2].

Multiple retrospective and prospective studies have demonstrated the safety and effectiveness of BAE. Immediate control of bleeding is achieved in approximately 85%-98% of cases [2], with long-term recurrence rates ranging from 20% to 45% depending on the underlying pathology and technical factors [3,4]. Most recurrences occur within the first two years, emphasizing the need for close postprocedure surveillance.

While BAE is generally well tolerated, minor complications such as transient chest pain, dysphagia, and fever may occur. Severe complications, including spinal cord ischemia, are rare when meticulous angiographic technique is employed. Current evidence suggests that embolization of nonbronchial systemic collaterals and early intervention following the onset of hemoptysis can improve outcomes [4].

Given these considerations, this study aims to evaluate the outcomes of BAE in patients presenting with moderate to severe hemoptysis, with a focus on immediate success, recurrence rates, etiology distribution, and complications. Understanding these aspects will contribute to refining selection criteria, improving procedural planning, and ultimately enhancing patient outcomes in both emergency and elective settings.

## Materials and methods

Study design

The present study is a cross-sectional longitudinal study conducted over 18 months (from July 2023 to January 2025) in the Department of Respiratory Medicine of Shri Guru Ram Rai Institute of Medical and Health Sciences, Dehradun, India. The study included cases from both the outpatient department and the inpatient department, as well as referrals from other departments.

Inclusion and exclusion criteria

The study included patients aged 18 and above, irrespective of gender, who presented with a clinical diagnosis of hemoptysis and provided informed written consent. Individuals were excluded if they had significant cardiac conditions such as recent myocardial infarction, valvular heart disease, or Eisenmenger syndrome. Other exclusion criteria included renal insufficiency, defined as a serum creatinine level greater than 1.5 mg/dL or an eGFR below 40, provided they were not planned for dialysis. Additionally, individuals with a known allergy to iodine-based contrast agents were also excluded.

Study endpoints

The study's primary endpoint was immediate clinical success, defined as complete cessation or marked reduction of hemoptysis within 24 hours following BAE. Secondary endpoints included recurrence of hemoptysis at one and three months after BAE, incidence and type of complications (both immediate and delayed), identification of common etiologies, and procedural outcomes based on the vessels embolized and associated clinical factors.

Embolization procedure details

**Figure 1:**
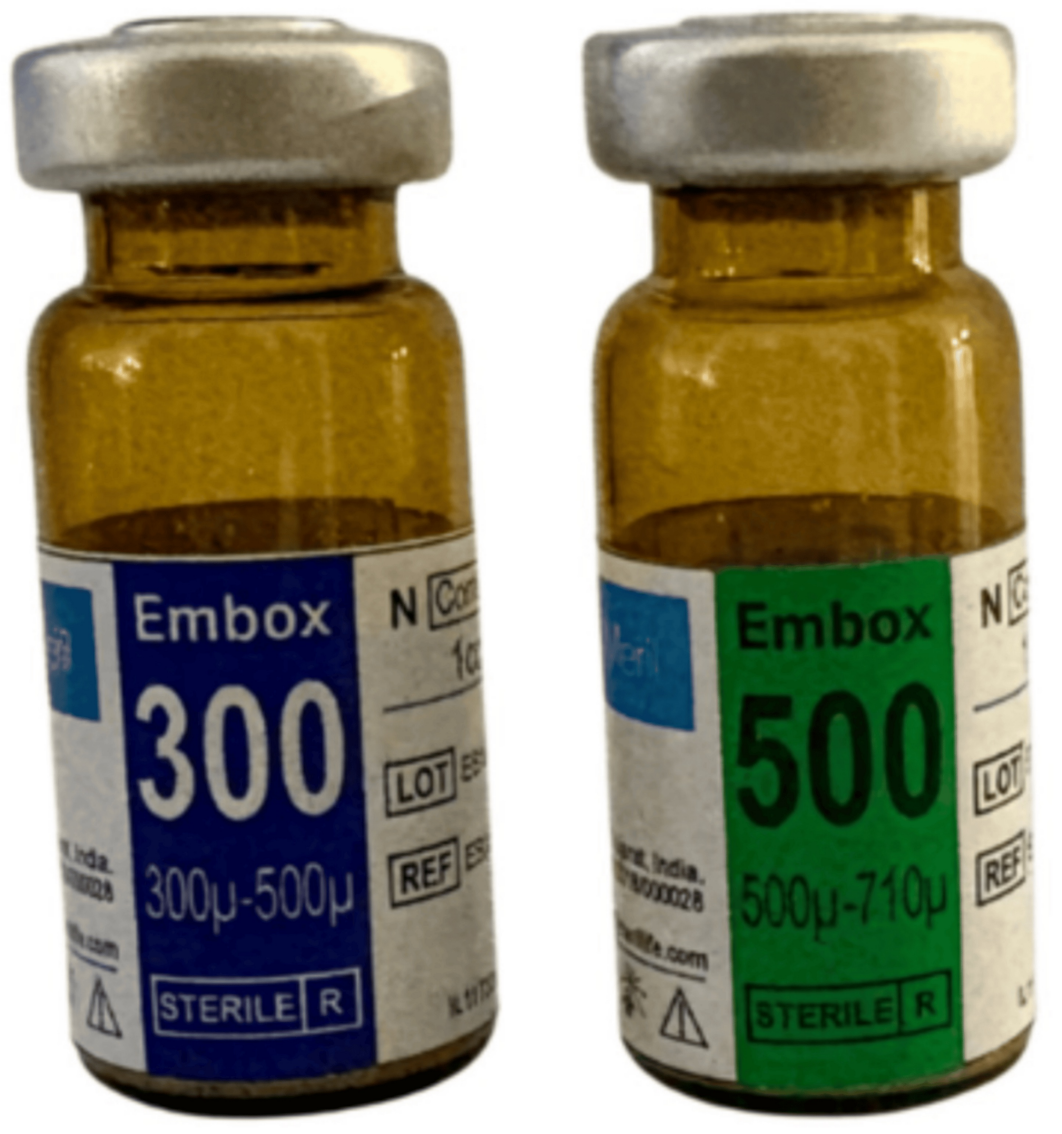
Embox 300 and 500 (Meril Life Sciences Pvt Ltd, Vapi, India)

BAE was performed using Embox (Meril Life Sciences Pvt. Ltd., Vapi, India) under local anesthesia using the common femoral artery approach via insertion of a 6 Fr vascular sheath. Conscious sedation was generally avoided due to the risk of respiratory depression in patients with compromised pulmonary function. An initial descending thoracic aortogram was performed using a C1 catheter and nonionic contrast agent to localize the bronchial arteries. Subsequent selective catheterization of the abnormal bronchial arteries was performed using a microcatheter and microwire coaxial system. The choice of vessels for embolization was guided by findings from prior contrast-enhanced CT scans and confirmed angiographically by features such as hypertrophied or tortuous arteries, focal hypervascularity, extravasation of contrast, bronchopulmonary shunting, or the presence of bronchial artery aneurysms. When nonbronchial systemic arteries were identified as contributors to the hemoptysis, they were also selectively embolized in the same sitting, depending on technical feasibility. Embolization was carried out using Embox, a PVA foam-based embolic agent. Particles measuring 300-500 microns were used for smaller vessels, while 500-710 micron particles were selected for larger vessels (Figure 1). The agent was administered slowly to minimize reflux and achieve controlled vascular occlusion. Repeat angiograms were performed intermittently to assess the adequacy of embolization and to identify any new or collateral vessels that may have emerged as distal branches became occluded.

Following the procedure, all patients were monitored in the intensive care unit for 24 hours for immediate postprocedural complications, including neurological assessments to rule out spinal cord ischemia. Patients were subsequently followed up at one and three months to evaluate for recurrence of hemoptysis and any delayed complications.

Sample size calculation

The sample size was calculated based on the assumption that 99% of patients with moderate to severe hemoptysis would undergo BAE as an effective intervention, based on findings from previous literature. Using this expected proportion, a confidence level of 99%, and an absolute precision of 2%, the minimum required sample size was estimated to be 179 patients. This calculation was performed using standard formulas for proportions in descriptive studies. To accommodate potential dropouts and ensure adequate power for subgroup analyses, a final sample of 203 patients was included in the study.

Statistical analysis

Microsoft Excel (Microsoft Corporation, Redmond, WA) and Statistical Package for the Social Sciences (version 27; IBM Corp., Armonk, NY) were used for data cleaning and analysis. Categorical and continuous data were reported as proportions and mean ± SD.

Ethical approval

Prior to the study, approval was obtained from the Institutional Ethics Committee (SGRR/IEC/19/23).

## Results

Demographics and baseline hemoglobin

The study cohort included a higher percentage of older adults, with 90 (44.3%) aged 60 years or older and 82 (40.4%) aged between 41 and 60 years. The male predominance was notable, comprising 137 participants (67.5%). Nutritional status, assessed by BMI, revealed that more than half were underweight (BMI < 18.5), 103 (50.8%), while 63 (31.1%) had a BMI of 18.5-22.9, and a minority, 13 (7.3%), had a BMI > 25. Baseline hemoglobin levels indicated that 106 (52.2%) of the participants had severe anemia (<7 g/dL), whereas 74 (36.4%) had hemoglobin levels between 7 and 9.9 g/dL (Table 1).

**Table 1:** Demographics and baseline hemoglobin

Variable	Frequency (%)
Age distribution	
18-40	31 (15.3)
41-60	82 (40.4)
>60	90 (44.3)
Gender distribution	
Male	137 (67.5)
Female	66 (32.5)
Body mass index	
<18.5	103 (50.8)
18.5-22.9	63 (31.1)
23-24.9	24 (11.8)
>25	13 (7.3)
Smoking	100 (49.3)
Baseline hemoglobin	
<7 g/dL	106 (52.2)
7-9.9 g/dL	74 (36.4)
10-11.9 g/dL	16 (7.8)
≥12 g/dL	7 (3.6)

Hemoptysis distribution and chest findings

Among the patients, 119 (58.6%) experienced moderate hemoptysis (250-500 mL), while 84 (41.4%) had massive hemoptysis (>500 mL). Chest imaging revealed right-sided involvement in 79 (38.9%), left-sided involvement in 57 (28.1%), and bilateral findings in 67 (33%). Field involvement was nearly equally distributed, with upper field involvement in 103 (50.7%) and lower field involvement in 100 (49.3%). Mild disease limited to one zone was noted in 99 (48.8%), moderate disease involving two zones on the same side in 35 (17.2%), and severe disease spanning three zones or bilateral sides in 69 (34%). Regarding disease patterns, the most common was cavity with aspergilloma, observed in 89 (43.8%), followed by cavitary bronchiectasis without aspergilloma in 48 (23.6%). Consolidation with cavitation and noncavitary bronchiectatic changes was seen in 15 (7.5%), and 20 (9.8%) of radiographs appeared normal (Table 2).

**Table 2:** Hemoptysis distribution and chest findings

Variables	Frequency (%)
Hemoptysis distribution	
Moderate (250-500 mL)	119 (58.6)
Massive (>500 mL)	84 (41.4)
Chest findings	
Right	79 (38.9)
Left	57 (28.1)
Bilateral	67 (33)
Field involvement	
Upper field	103 (50.7)
Lower field	100 (49.3)
Extent of disease	
Mild (one zone)	99 (48.8)
Moderate (two zones of the same side)	35 (17.2)
Severe (three zones and bilateral side)	69 (34)
Disease pattern	
Consolidation with cavitary lesion (infective)	15 (7.5)
Noncavitary disease with bronchiectasis	15 (7.5)
Cavitary disease with bronchiectasis without aspergilloma	48 (23.6)
Cavity with aspergilloma	89 (43.8)
Mass (well-defined opacity)	16 (7.8)
Normal	20 (9.8)

Truenat and 2D echo distribution of patients

Truant testing for *Mycobacterium tuberculosis *(MTB) revealed that MTB was detected in 15 (7.4%) of patients, while 188 (92.6%) had no detectable MTB. Echocardiographic evaluation showed Cor pulmonale features indicative of right heart failure in 91 (44.8%) of patients, whereas 112 (55.2%) exhibited normal cardiac findings (Table 3).

**Table 3:** Truenat and 2D echo distribution of patients MTB: *Mycobacterium tuberculosis*; 2D: two dimensional

Variable	Frequency (%)
MTB	
MTB not detected	188 (92.6)
MTB detected	15 (7.4)
2D echo	
Cor pulmonale (right heart failure)	91 (44.8)
Normal	112 (55.2)

Diagnosis, vessel involvement, and outcome

Among the patients evaluated, aspergilloma was the most common diagnosis, noted in 89 (43.8%), followed by bronchiectasis in 39 (19.2%) and fibrocavitary lesions in 24 (11.8%). Malignancy was observed in 16 (7.9%), while 15 (7.4%) had nonspecific post-tuberculosis pulmonary changes, and 20 (9.9%) were categorized as idiopathic. The most frequently involved vessel was bilateral bronchial arteries, 64 (27.8%), followed by common bronchial, 37 (16.1%), left bronchial, 34 (14.8%), and right bronchial arteries, 29 (12.6%). Other vessels included the intercostobrachial trunk, 24 (10.4%), internal mammary artery, 14 (6.1%), posterior intercostal artery, 12 (5.2%), costocervical trunk, 11 (4.8%), and thyrocervical trunk, 5 (2.2%). Regarding clinical outcomes, 151 (74.3%) patients achieved treatment success, 22 (10.8%) experienced treatment failure, 23 (11.3%) were lost to follow-up, and seven (3.4%) succumbed during the study period (Table 4).

**Table 4:** Diagnosis, vessel involvement, and outcome NSPPTB: nonspecific post-tuberculosis pulmonary changes

Variable	Frequency (%)
Diagnosis	
Aspergilloma	89 (43.8)
Bronchiectasis	39 (19.2)
Fibrocavitatory lesions	24 (11.8)
Malignant	16 (7.9)
NSPPTB	15 (7.4)
Hemoptysis cause idiopathic	20 (9.9)
Vessel involvement	
Intercostobrachial trunk	24 (10.4)
Right bronchial	29 (12.6)
Left bronchial	34 (14.8)
Common bronchial	37 (16.1)
Bilateral	64 (27.8)
Posterior intercoastal artery	12 (5.2)
Internal mammary artery	14 (6.1)
Thyrocervical trunk	5 (2.2)
Costocervical trunk	11 (4.8)
Outcome	
Death	7 (3.4)
Treatment failure	22 (10.8)
Treatment success	151 (74.3)
Lost to follow-up	23 (11.3)

Complications and follow-up data

Immediate postprocedural complications most commonly included chest pain in 119 (58.6%) patients, followed by fever in 30 (14.8%), headache in 24 (11.8%), and streaking hemoptysis in 19 (9.4%). Neuropathy occurred in one (0.5%), while immediate mortality was observed in three (1.5%), and treatment failure in seven (3.4%).

At the one-month follow-up, chest pain remained prevalent in 98 (55.7%), with fever in 37 (21.0%), headache in 22 (12.5%), and streaking hemoptysis in 17 (9.7%). Neuropathy was reported in two (1.1%), mortality in three (1.7%), treatment failure in 10 (5.7%), and 12 (6.8%) patients were lost to follow-up.

**Figure 2:**
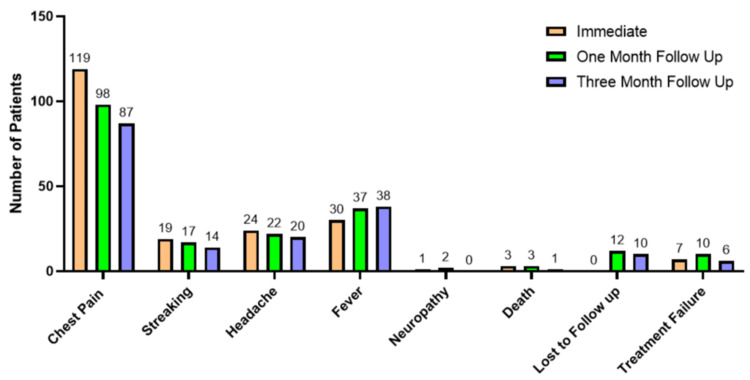
Complications and follow-up data

At three months, chest pain persisted in 87 (54.7%), followed by fever in 38 (23.9%), headache in 20 (12.6%), and streaking hemoptysis in 14 (8.8%). No neuropathy cases were reported at this time; mortality decreased to one (0.6%), treatment failure was observed in six (3.8%), and 10 (6.3%) patients were lost to follow-up (Table 5 and Figure 2).

**Table 5:** Complications and follow-up data

Variable	Frequency (%)
Immediate	
Chest pain	119 (58.6)
Streaking	19 (9.4)
Headache	24 (11.8)
Fever	30 (14.8)
Neuropathy	1 (0.5)
Death	3 (1.5)
Lost to follow-up	0 (0)
Treatment failure	7 (3.4)
One-month follow-up	
Chest pain	98 (55.7)
Streaking	17 (9.7)
Headache	22 (12.5)
Fever	37 (21)
Neuropathy	2 (1.1)
Death	3 (1.7)
Lost to follow-up	12 (6.8)
Treatment failure	10 (5.7)
Three-month follow-up	
Chest pain	87 (54.7)
Streaking	14 (8.8)
Headache	20 (12.6)
Fever	38 (23.9)
Neuropathy	0 (0)
Death	1 (0.6)
Lost to follow-up	10 (6.3)
Treatment failure	6 (3.8)

## Discussion

Our study cohort had a predominance of older adults, with 44.3% aged over 60 years, and a clear male predominance (67.5%). Similar demographic patterns have been observed in other large series on hemoptysis in tuberculosis-endemic regions, where elderly males tend to be disproportionately affected due to chronic lung diseases and long-standing tobacco use [5,6]. Nutritional status was notably compromised in our patients, with over half being underweight (BMI < 18.5) and more than 50% presenting with severe anemia (Hb < 7 g/dL). These indicators reflect systemic depletion and chronic inflammation, often observed in post-tuberculosis (post-TB) sequelae [7].

Moderate to massive hemoptysis was reported in all patients, with 41.4% presenting with massive hemoptysis. This rate is comparable to that reported by Swanson et al. and Jean-Baptiste, who documented similar figures in life-threatening cases [8,9]. Radiologically, upper and lower field involvement were equally distributed, and bilateral lung involvement was seen in one-third of the cases, consistent with findings in postinfectious bronchiectasis and mycetoma patients [10]. Cavity with aspergilloma (43.8%) was the predominant radiologic pattern, aligning with prior studies highlighting its high bleeding potential [11,12].

Only 7.4% of cases were positive for MTB by Truenat, affirming that active TB is not the leading cause of hemoptysis in this population. Instead, chronic sequelae dominate, as shown in similar studies where post-TB bronchiectasis and fibrocavitary disease were the major contributors [5]. Notably, 44.8% of patients had echocardiographic evidence of cor pulmonale, supporting previous findings linking chronic lung pathology with right heart strain in hemoptysis patients [13].

Aspergilloma and bronchiectasis were the leading diagnoses. Bilateral bronchial arteries were the most commonly involved vessels (27.8%), necessitating the embolization of multiple arteries, a finding corroborated by Woo et al. [14] and Panda et al. [6]. The overall success rate of BAE in our study was 74.3%, which, although slightly lower than international reports of 80%-90%, is reasonable considering the complexity and chronicity of cases [15].

The most frequent immediate complications were chest pain (58.6%) and fever (14.8%), both of which were self-limiting and consistent with earlier reports by Yoon et al. [10] and Mal et al. [16]. Clinical improvement was sustained in most patients at one- and three-month follow-ups, with treatment failure reported in only 5.7% and 3.8%, respectively. Mortality remained low, with just 0.6% mortality at three months. These outcomes reaffirm BAE’s role as an effective short- to mid-term intervention in managing massive hemoptysis.

Additional comparative findings from global literature strengthen these observations. In a study by Chun and Belli, BAE had a success rate of 86%, with recurrence in 28% of cases [17]. This is pertinent to our cohort, where multiple comorbidities may have influenced outcomes.

Despite technical challenges, our low rate of major complications, including a single case of neuropathy (0.5%), compares favorably with international safety benchmarks [15]. This reflects skilled catheter technique and rigorous angiographic screening.

Strength of the study

This study’s key strength lies in being one of the first regionally conducted investigations to provide comprehensive longitudinal data on BAE outcomes in a tuberculosis-endemic population. Including a large sample size (203 patients) strengthens the generalizability of findings, particularly for clinical settings dealing with post-tubercular lung damage. Additionally, the study’s systematic approach covering imaging, etiology, procedural details, and short-term follow-up offers a robust dataset for clinical interpretation.

Limitations of the study

The single-center observational design limits the external validity of the results. The follow-up period of only three months may have missed longer term recurrences or late complications, and the 11.3% loss to follow-up may have introduced bias in outcome evaluation. Furthermore, the absence of a control or comparative treatment group restricts the ability to evaluate BAE against surgical or conservative modalities directly.

## Conclusions

This study reinforces the role of BAE as a frontline intervention in managing moderate to severe hemoptysis. With an immediate clinical success rate of over 74% and manageable complication rates, BAE proves to be an effective and safe alternative to surgery in high-risk or medically complex patients. The predominance of aspergilloma and bronchiectasis among etiologies also highlights the ongoing burden of post-tuberculosis lung damage in endemic regions. Long-term follow-up and careful patient selection remain key to optimizing outcomes and reducing recurrence.
